# Supplementary material for: Origin and Genealogy of Rare mtDNA Haplotypes Detected in the Serbian Population
Source: Genes (Basel). 2025 Jan 20;16(1):106. doi: 10.3390/genes16010106 (PMC11765032; doi:10.3390/genes16010106)
Supplement: Supplementary file 1 [file genes-16-00106-s001.zip › Supplementary RESULTS.pdf]

SUPPLEMENTARY RESULTS

Origin and genealogy of rare mtDNA haplotypes detected in the Serbian population

Slobodan Davidović, Jelena M. Aleksić, Marija Tanasković, Pavle Erić, Milena Stevanović, Nataša Kovačević-Grujičić

Contents

Subhaplogroup R0a 2
Subhaplogroup N1a 2
Subhaplogroup N1b 5
Subhaplogroup I5 6
Haplogroup W 6
  Subclade W1 6
  Subclade W1c 7
  Subclade W1h and W1e1 7
  Subclade W3 7
  Subclade W5 8
Subhaplogroup X2 8
  Subclade X2 8
  Subclade X2b+226 9
  Subclade X2m'n, X2m and X2n 9
Supplementary Figures 10
  Figure S7. 10
  Figure S9. 11
  Figure S11. 12
  Figure S15. 13
  Figure S16. 14
  Figure S18. 15
  Figure S20. 16
References: 17

Subhaplogroup R0a

The frequency distribution of subhaplogroup R0a suggests the direction of migrations of the carriers of this lineage from the Near East towards the South European peninsulas (Figure 1 and Table S3). The regions with the most haplotypes (Hp) belonging to subclade R0a were the Near East and Africa followed by the Apennine and Balkan Peninsulas (Table S2). On the other hand, the highest haplotype diversity (HD) parameter was detected in the Apennine Peninsula followed by the Near East (Table S2). Interestingly, high values of this parameter were also detected in Africa and the Balkan Peninsula. These results indicate that the migration routes from the Near East to Europe went through the Apennine and the Balkan Peninsulas. The lowest value for the random match probability parameter (RMP) was detected for the Near East and the Apennine Peninsula (Table S2), suggesting that these regions are the most diverse.

The ancestral HVS-I/HVS-II haplotype from which the haplotype detected in the Serbian population (98_Sb) diverges was found in 22 individuals mainly originating from the Balkan (nine individuals) and the Arabian Peninsula (seven individuals), but also from Central Europe (three individuals), the Apennine and the Iberian Peninsulas, and Northern Africa (one individual each) (Figure 2 and Table S3). This group of haplotypes differs from a more frequent R0a ancestral haplotype by a transition at np 152. The ancestral R0a haplotype that gave rise to 98_Sb and other haplotypes positioned in its branch is present in the Balkan Peninsula and other European regions, even though it is the most frequent in the Near East suggesting that the ancestral HVS-I/HVS-II haplotypes of the one detected in sample 98_Sb were most likely present in Southern Europe before it diverged 1.54-1.8 kya. These ancestral haplotypes could have arrived in the Balkan Peninsula during the Hellenistic period after Alexander the Great’s conquests, or the Roman Empire’s period when this part of Europe was under the strong influence or direct control of ancient states. Interestingly, the highest frequency of subhaplogroup R0a can be detected on the territory of modern-day Greece (Figure 1 and Table S3).

Subhaplogroup N1a

The frequency distribution of subhaplogroup N1a in modern-day populations of Europe and the Near East supports the findings that it arrived in Europe during the Neolithic expansion (Figure 1, Table S4). According to our data, the highest HD values were detected in Asia and the Near East (Table S2). Additionally, the highest values for the parameters nucleotide diversity (ND) and the

mean number of pairwise differences (MPD) were detected for the Apennine Peninsula, Asia, and the Near East, which shows that haplotypes detected in these regions were more diverse among themselves than the haplotypes in other regions for which we have detected similar HD values. The Balkan Peninsula has somewhat lower ND and MPD values than the previously mentioned regions (Table S2). The lowest RMP value was detected for the Near East, followed by Central Europe and the Balkan Peninsula (Table S2) suggesting these regions are the most diverse.

The HVS-I/HVS-II haplotype found in sample 154_Sb was identical to 21 haplotypes detected in Eastern Europe (three haplotypes from Russia and Estonia each), Northern Europe (five from Finland and one from Denmark), Central Europe (two from the Czech Republic and one from Slovakia), and the Balkan Peninsula (four from Bulgaria), as well as one from Asia and one ancient haplotype from Bulgaria (BM 36, [1]) dated to the Early Bronze Age (Table S4, Figure 3). The presence of this HVS-I/HVS-II haplotype in the Balkan Peninsula during the Bronze Age suggests that it could have arrived during the Bronze Age migrations of the Yamnaya herders [1,2]. Interestingly, the haplotype detected in the ancient sample HAL4 from Germany [2] dated to around 5 kya, which differs from the 154_Sb haplotype by a transition at np 152, suggests that these lineages were already present in Central Europe during the Neolithic. It is possible that some of the 16,147A branch lineages arrived in the Balkan Peninsula during the Neolithic expansion and later during the Bronze Age migrations of the carriers of Yamnaya culture.

Using complete mitogenomes to reconstruct the phylogeny of subhaplogroup N1a we have identified three new subclades: N1a1a1a1b, N1a1a1a1c, and N1a1a1a1d. The estimated age of the subclade N1a1a1a1b is 2.71 ky and it clusters haplotypes from Serbia (242_Sb) and Russia (Figure S2). Subclade N1a1a1a1c consists of haplotypes from Slavic populations (Russian, Polish, and Croatian) with an estimated age of 1.35-2.31 kya (Figure S2). The N1a1a1a1d subclade contains haplotypes from Estonia and Poland with the proposed age of 7.33-10.51 kya. Three haplotypes from ancient remains are classified into the N1a1a1a1 subclade as well: Anc4 [3] and LHSZ27B [4] both excavated in modern-day Hungary, and RISE391 excavated in Kazakhstan [5]. The grave of the sample Anc4 was dated to the mid-10th century of the Common Era (CE) and was typical of classical Hungarian conquerors who arrived in the Danube basin at the end of the 9th century CE [6]. These people were known as Magyars and originally came from the region around the Ural Mountains [6]. The sample LHSZ27B originates from the 6th century CE grave belonging to the

Longobard culture i.e., Germanic people who arrived in the territory of the Pannonian Plain during the Migration Period [4,7]. The haplotypes of samples Anc4 and LHSZ27B differentiate from the 154_Sb haplotype by the transitions at nps 228 and 16,195, respectively (Figures 3 and S2). The HVS-I/HVS-II haplotype identical to the LHSZ27B sample was detected in the individual from Northwest Germany [8] (Table S4). The haplotype found in ancient sample RISE391, belonging to the Sintashta culture (2100-1800 BCE), differentiates from the haplotype detected in the sample 154_Sb by the transition at the np 16,189 (Figure 3 and Table S4).

Based on HVS-I/HVS-II variation, four N1a haplotypes, detected in the Serbian population are defined by 16,147G transversion (33_Sb [9], VP87 [10], Ser_03 [11], and 232_Sb [9]) (Figure 3, Table S4). Within the 16,147G branch, besides the Near East and African haplotypes, we could detect 15 European haplotypes predominantly found in the Balkan Peninsula (Figure 3). In the Balkan Peninsula, the 16,147G branch is present mostly in the Slavic-speaking populations (Serbia, Bosnia and Herzegovina, Croatia, and Bulgaria) and occasionally in the Greek population (Table S4). Within the N1a phylogeographic network, one of the 16,147G haplotypes was found in the individual who lived during the Early Bronze Age around 4 kya in the territory of modern-day Russia near Volgograd (RISE555, [5]). Interestingly, this HVS-I/HVS-II haplotype, defined by a transition at the np 16,344, is identical to the one found in the Near East (Table S4 and Figure 3). Nevertheless, the phylogeny reconstruction using complete mitogenomes showed that these two haplotypes differ from each other forming a new subclade N1a1a4 defined by the transition at np 8452 with an estimated age between 12.07-13.83 kya (Figure S3). This subclade also includes one more haplotype from the Caucasus region suggesting that the N1a1a4 subclade could have spread into Eastern Europe through the Caucasus. Further expansion toward the Balkan Peninsula might have occurred during the Bronze Age with the carriers of Yamnaya culture [2,5] and by Slavic migrations during the Migration Period in the Early Middle Ages. Another possibility is that this subclade reached the Balkans through Asia Minor, where today a 16,147G branch is the most frequent in Europe. Afterwards, it could have spread towards Eastern Europe where it was detected in the Bronze Age sample RISE555.

In the N1a phylogeographic network, a new branch that has back mutation at np 16,147 can be identified (Serbian samples 242_Sb [9] and Nish8 [12]) (Figure 3). This branch is most common in the populations of the Near East while in Europe it is present in the Apennine (seven haplotypes

in nine individuals) and Balkan Peninsulas (six haplotypes found in the same number of individuals) (Table S4 and Figure 3). Interestingly, two out of three haplotypes from Central Europe originate from Hungary and Romania which border the Balkan Peninsula while only two haplotypes were found in Northern Europe and the Iberian Peninsula (Table S4, Figure 3). Considering the arrival of the N1a subhaplogroup in Europe with the Neolithic package [13], this branch might have arrived during the Neolithic expansion in Southeastern Europe (the Balkan and Apennine Peninsula) later spreading further into the continent.

Subhaplogroup N1b

The lowest value for the parameter RMP was detected for the Near East and the Balkan Peninsula (Table S2) suggesting that these regions are the most diverse.

Within the 16,176A branch, a HVS-I/HVS-II haplotype ancestral to the ones detected in Serbian samples Nish9 and 143_Sb, we identified two haplotypes found in Longobard individuals who lived in Pannonia plain in the 6th century CE (grave 22, [14] and sample LHSZ13 [4], and one in the Bronze Age individual who lived in Southwestern Germany 2029-1892 BCE (sample MX275MT, [15]) (Table S5, Figure 4). Two additional haplotypes in the 16,176A branch found in ancient remains were from another Longobard individual from the 6th century CE excavated in the Czech Republic (LRCMUS69, [4]) and one sample from France dated to 2463-2208 BCE (SX32MT, [15]) (Table S5, Figure 4). Interestingly, most of the HVSI/HVS-II haplotypes of Near Eastern origin belonging to the 16,176A branch are found in the Ashkenazi Jewish population (Table S5 and Figure 4). Ashkenazi Jews have admixed origin and share to a great extent ancestry with the South European populations [16-18].

Three HVSI/HVS-II haplotypes from the Serbian population that belong to the branch defined by the transition at np 152 are further characterized by transitions at the nps 146 and 16,244 putting them in the European branch. This branch included haplotypes mostly from Romania (11 individuals) and the Balkan Peninsula (four individuals) while one haplotype was found in Germany and two in Sardinia (Table S5 and Figure 4).

Several new subclades were identified after phylogeny reconstruction using complete mitogenomes: N1b1a7a, N1b1a10, N1b1a11, N1b1a13, N1b1a14, and N1b1a15 (Figure S5).

Subhaplogroup I5

The lowest values for the parameter RMP were detected for the Balkan Peninsula and Central Europe (Table S2) suggesting these regions are the most diverse.

HVS-I/HVS-II haplotype ancestral to the 16,391 branch with the Serbian haplotype contains a haplotype detected in the ancient individual from the late 10th century CE excavated in modern-day Hungary (Table S6, Figure 5). This individual (sample Anc10 [3]) was identified as a commoner ie. a member of the population who inhabited the Pannonia Plain before the settlement of Magyar tribes in the late 9th century CE [6]. This ancestral haplotype was also detected in the Near East (Druze and Bedouin), the Caucasus (Armenia), Italy, the Balkan Peninsula, and North Africa (Jews) (Table S6).

Phylogeny reconstruction based on complete mitogenomes showed that entire I5a1 clade clusters haplotypes of predominantly European origin suggesting that this old subhaplogroup could have evolved in Europe (Figure S5).

Haplogroup W

The highest values of the parameter Hp were detected in Central Europe followed by the Near East, the Apennine, and the Balkan Peninsulas (Table S2). Most of the analyzed regions displayed high values of parameter HD. The highest HD value was detected in the Apennine Peninsula and the lowest value was observed in Northern Europe (Table S2). High values for the parameters ND and MPD were detected in the Balkan and the Apennine Peninsulas, Central Europe, Asia, and the Near East (Table S2). The lowest values for the parameter RMP were detected for Central Europe and the Apennine Peninsula (Table S2) suggesting that these regions are the most diverse.

Subclade W1

Based on the complete mitogenome phylogeny, we identified new subclades: W1j, W1k, W1l, W1m, and W1m (Figure S8). Newly defined subclades W1k and W1l are defined with transition at np 143 putting them in the paragroup together with another novel subclade W1j of probable South European origin. W1k subclade whose age estimate is between 3.39-3.46 kya clusters four haplotypes detected in Eastern Europe suggesting that it could have evolved in this region during

the late Bronze Age. On the other hand, subclade W1l (1.97-3.46 kya) is confined to the Apennine Peninsula (Sardinia).

Subclade W1c

Within subhaplogroup W1c, we have detected HVS-I/HVS-II haplotypes found in three early Neolithic individuals excavated in Germany belonging to Schöningen culture (SALZ 19, SALZ 20, SALZ 35, [19]), two Neolithic individuals from Funnel Beaker culture who all lived around 5 kya (poz461 and poz471, [20]) and one from the Corded Ware culture who lived around 4.5 kya on the territory of modern-day Poland (poz483, [20]) (Table S7 and Figure S9).

Phylogeny reconstructed with complete mitogenomes allowed us to identify several new subclades: W1c2, W1c3, W1c4, W1c5, W1c6, and W1c7 (Figure S10). The newly defined subclade W1c3 clusters two Polish haplotypes, one belonging to the previously mentioned Corded Ware culture individual (poz483, [20]) (Figure S10). This aligns with the time estimate of 4.08-4.61 kya indicating that this lineage might have evolved in Central Europe. Subclade W1c5 gathers haplotypes found in Asia (Iran and India), while subclades W1c6 and W1c7 cluster haplotypes from the Danish population (Figure S10).

Subclade W1h and W1e1

HVS-I/HVS-II haplotypes detected in two ancient samples were classified in the W1e1 subclade (Table S7 and Figure S7). The haplotype found in sample TU471 from Finland [21], dated 1042-1248 CE, is ancestral to the Serbian haplotype (sample Nish4) (Figure S7). This haplotype is presently found in Northern Europe and the Apennine Peninsula (Table S7 and Figure S7). Another ancient haplotype (sample LE134mt, [22]) from England was dated to the medieval period (first half of the 14th century CE) belonging to a victim of the Black Death. It is three mutation steps away from sample Nish4 (Figure S7).

Subclade W3

Phylogeny reconstruction based on the complete mitogenomes allowed us to identify new subclade W3a1d2 containing haplotypes from Siberia (Figure S12).

We defined several new subclades by phylogeny reconstruction using complete mitogenomes: W3b2, W3b3, W3b4, W3b5, and W3b6 (Figure S13). Subclade W3b4 gathers haplotypes from South Asia and most likely evolved in this region, while subclade W3b6 clusters two haplotypes from the Near East.

Subclade W5

The frequent and widespread HVS-I/HVS-II haplotype detected in samples 27_Sb and Brestovac11 was also found in two individuals from Bronze Age Unetice culture who lived 3.6 kya on the territory of modern-day Germany (QLB39 and QLB41, [19]), an individual from Yamnaya culture dated to 2882–2698 BCE found in modern-day Ukraine (poz208, [23]) and two individuals from Southwestern Germany who lived during the Bronze Age, 2135-1961 BCE (MX270 and MX275, [15]) (Table S7 and Figure S11).

Three new subclades were identified based on complete mitogenome phylogeny: W5c, W5d, and W5e (Figure S14). Subclade W5c contains two haplotypes found in Central and Northern Europe while subclade W5d also includes a haplotype from the Near East (Figure S14). Subclade W5e clusters one haplotype from Poland and one haplotype found in the remains of the individual who lived in the 5th century BCE in Sardinia [24] (Figure S14 and Table S7). This finding aligns with the age estimate of the subclade W5e which is 2.31-2.71 kya.

Subhaplogroup X2

The highest values for the ND and MPD parameters were detected in Central Europe and the Balkan Peninsula (Table S2). The lowest value for the parameter RMP was observed in the Balkan Peninsula which was closely followed by the Near East (Table S2) suggesting that these regions are the most diverse.

Subclade X2

Through the phylogeny reconstruction using complete mitogenomes, we defined two new subclades. Subclade X2q2 of probable Near Eastern origin (6.92-8.27 kya) and X2q3 of putative South European origin (6.86-11.53 kya) (Figure S17).

Subclade X2b+226

The HVS-I/HVS-II haplotype detected in sample 36_Sb is one of Europe's most frequent X2b haplotypes. It was also observed in five ancient individuals: Catalhoyuk_20374 [25], POST_84 [26], POST_85 [26], LICOL36 [4], and ESP22 [2] (Figure S18). Remains excavated in the Catalhoyuk archaeological site in Turkey were dated to the Neolithic period between 6450 and 6380 BCE and represent the oldest remains having X2b haplotype [25]. The samples POST_84 and POST_85 belong to the ancient remains from Germany dating to the Early Bronze Age between 2120-1928 BCE [26]. The sample ESP22, dated 2454-2291 BCE, was also excavated in Germany and belonged to the late Neolithic Corded Ware culture [2]. LICOL36 was the youngest of the ancient remains from the 6th-7th century CE excavated in Italy from the grave that belonged to Longobard culture [4]. One ancient sample from Sicily, dated to the Bronze Age between 1500-1200 BCE (T73-M73-1, [27]) has a haplotype that is only one mutation step away (transition at the np 16,311) from the ancestral haplotype found in sample 36_Sb (Figure S18 and Table S8).

When reconstructing the phylogeny of subhaplogroup X2b using complete mitogenomes, we observed a great number of ancient haplotypes belonging to this subhaplogroup and its subclades (Figure S19). This analysis identified new subclades: X2b14, X2b16, and X2b18 (Figure S19). Newly proposed subclade X2b17 clusters only the haplotypes detected in ancient remains from Late Neolithic Switzerland, Bronze Age Sicily, and medieval England [15,22,27].

Subclade X2m'n, X2m and X2n

Phylogeny reconstructed using complete mitogenomes allowed us to identify several new subclades: X2m1a (7.88-9.22 kya), X2m2a (1.35-3.94 kya), and X2m2b (6.92-9.68 kya) (Figure S21). Subclade X2m1a seems to have a regional distribution specific to Southern Europe while other subclades cluster haplotypes of various geographical origins.

Supplementary Figures

Figure S7.

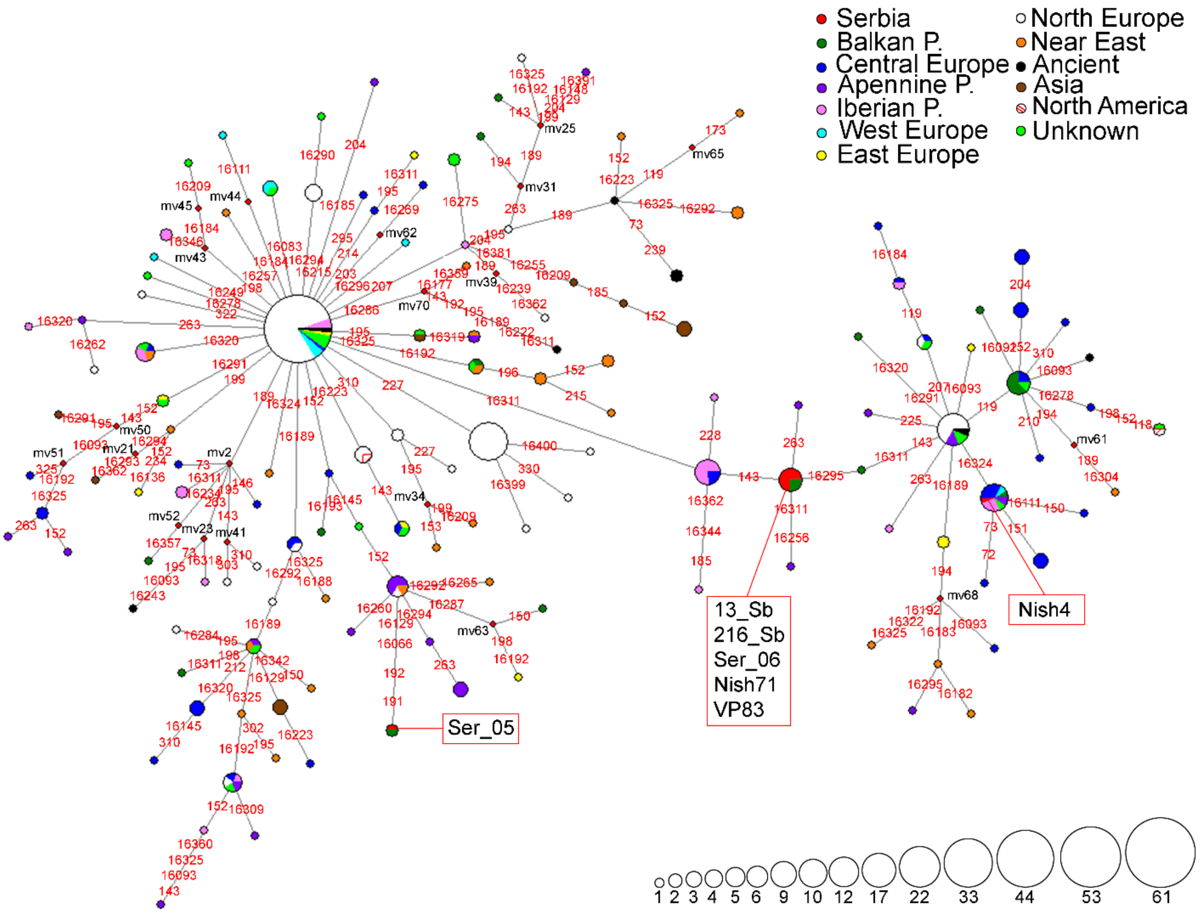

Figure S7. Part of the phylogeographic network for the W haplogroup (containing W1h and W1e1 subclades) based on 480 HVS-I/HVS-II haplotypes detected in 1022 individuals. Differences from rCRS are marked with red numbers representing nucleotide positions where the transitions occurred. Red rhomboids are the hypothetical haplotypes not detected in the analyzed sample (marked with mv). The sizes of the circles are proportional to the number of detected haplotypes as depicted in the legend. The geographical origin of the samples is presented in the legend.

Figure S9.

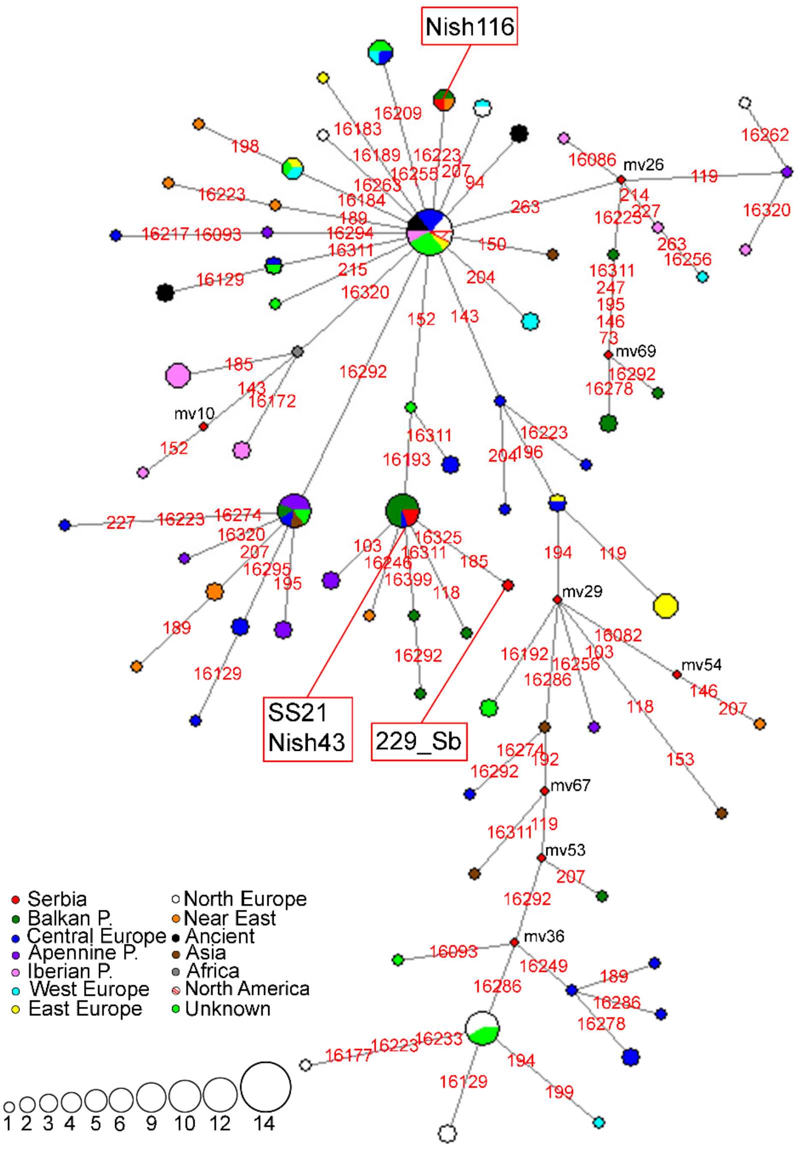

Figure S9. Part of the phylogeographic network for the W haplogroup (containing the W1c subclade) based on 480 HVS-I/HVS-II haplotypes detected in 1022 individuals. Differences from rCRS are marked with red numbers representing nucleotide positions where the transitions occurred. Red rhomboids are the hypothetical haplotypes not detected in the analyzed sample (marked with mv). The sizes of the circles are proportional to the number of detected haplotypes as depicted in the legend. The geographical origin of the samples is presented in the legend.

Figure S11.

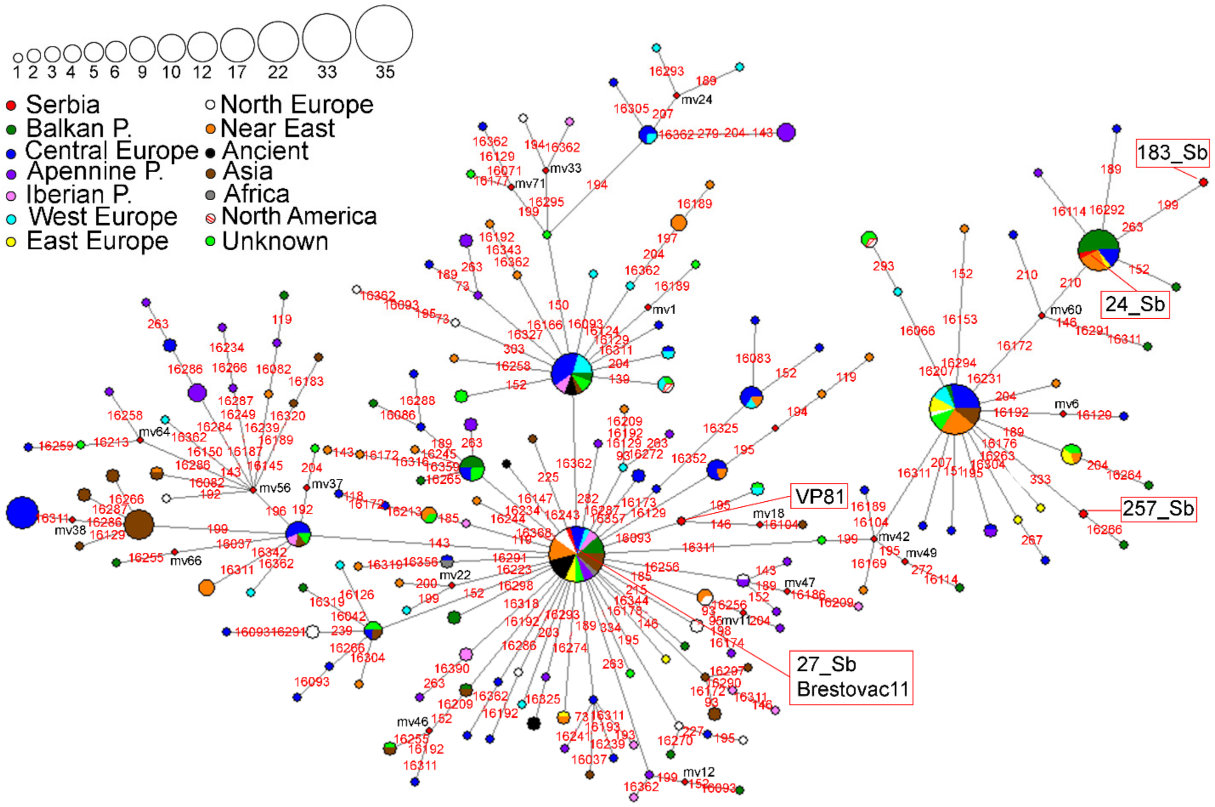

Figure S11. Part of the phylogeographic network for the W haplogroup based on 480 HVS-I/HVS-II haplotypes detected in 1022 individuals which contains the W3b’a subclade and other W subclades that cannot be classified based solely on HVS-I/HVS-II data. Differences from rCRS are marked with red numbers representing nucleotide positions where the transitions occurred. Red rhomboids are the hypothetical haplotypes not detected in the analyzed sample (marked with mv). The sizes of the circles are proportional to the number of detected haplotypes as depicted in the legend. The geographical origin of the samples is presented in the legend.

Figure S15.

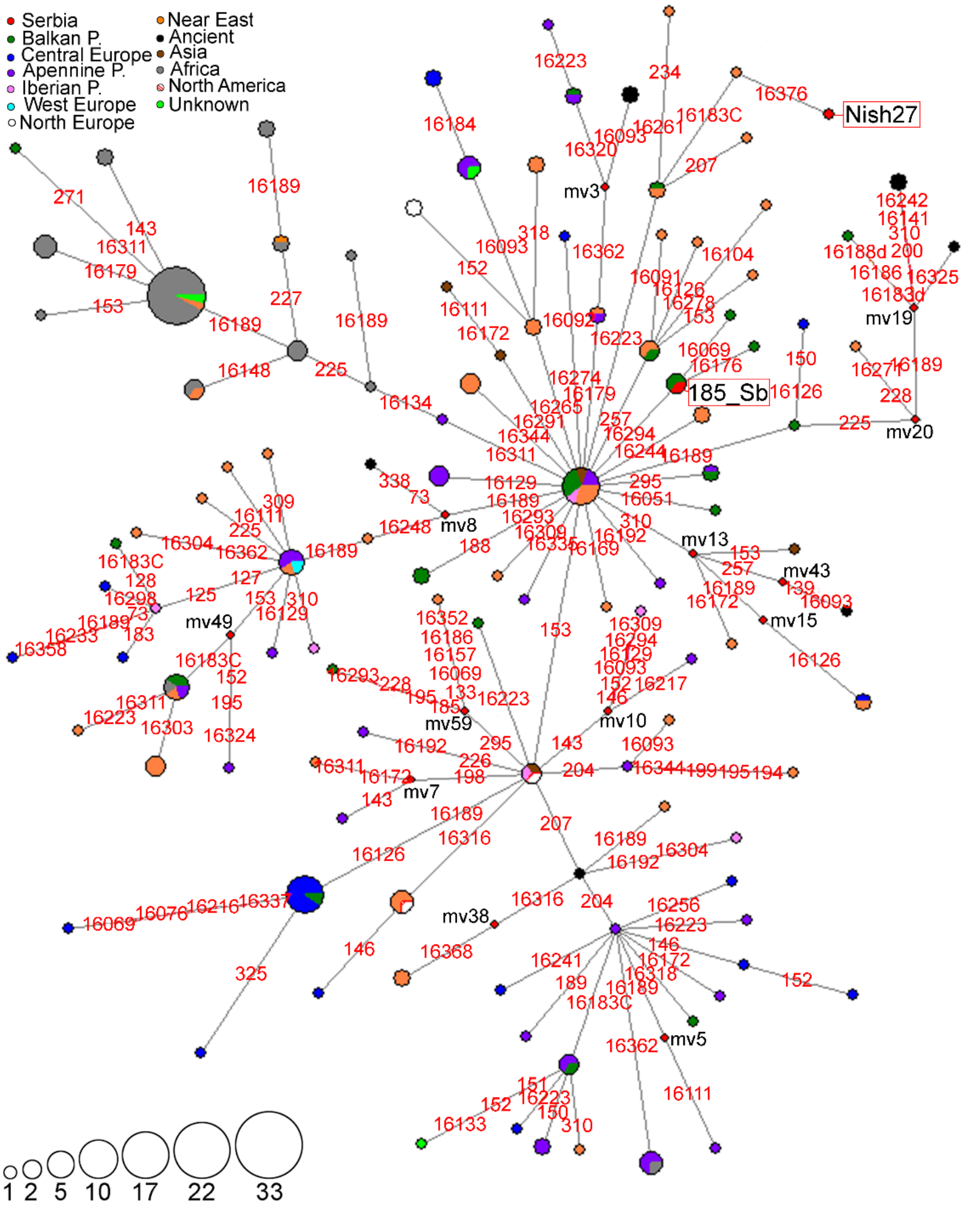

Figure S15. Part of the phylogeographic network for the X2 subhaplogroup based on 458 HVS-I/HVS-II haplotypes detected in 969 individuals. Differences from rCRS are marked with red numbers representing nucleotide positions where the transitions occurred. Red rhomboids are the hypothetical haplotypes not detected in the analyzed sample (marked with mv). The sizes of the circles are proportional to the number of detected haplotypes as depicted in the legend. The geographical origin of the samples is presented in the legend.

Figure S16.

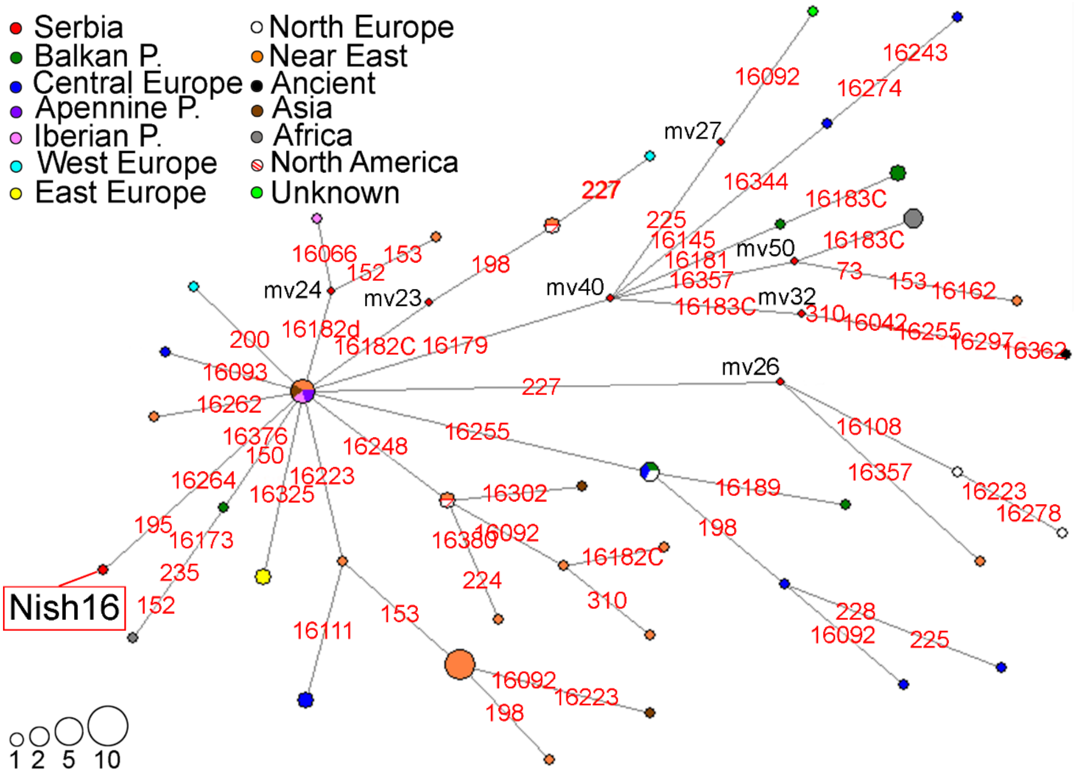

Figure S16. Part of the phylogeographic network for the X2 subhaplogroup based on 458 HVS-I/HVS-II haplotypes detected in 969 individuals. Differences from rCRS are marked with red numbers representing nucleotide positions where the transitions occurred. Red rhomboids are the hypothetical haplotypes not detected in the analyzed sample (marked with mv). The sizes of the circles are proportional to the number of detected haplotypes as depicted in the legend. The geographical origin of the samples is presented in the legend.

Figure S18.

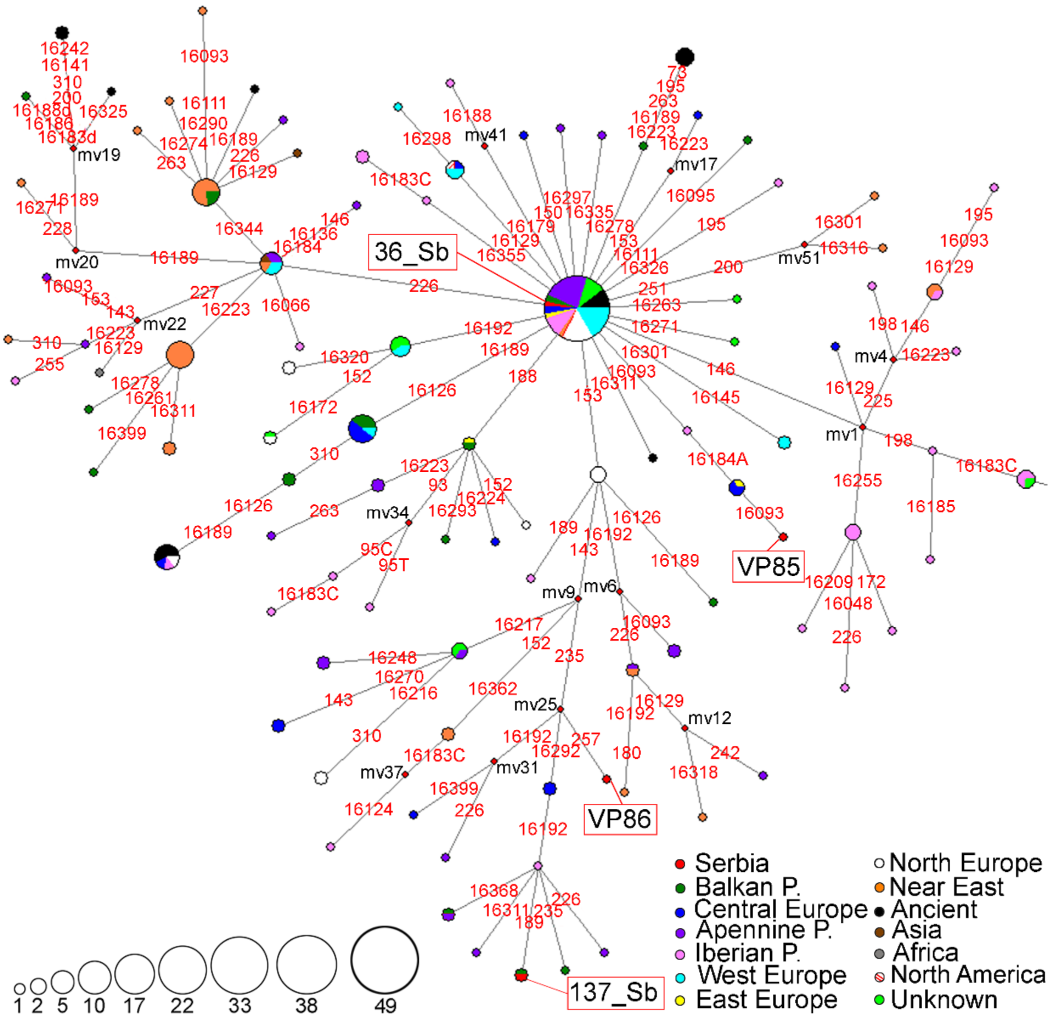

Figure S18. Part of the phylogeographic network for the X2 subhaplogroup based on 458 HVS-I/HVS-II haplotypes detected in 969 individuals which represents the X2m subclade and other X2 subclades that cannot be classified based solely on HVS-I/HVS-II data. Differences from rCRS are marked with red numbers representing nucleotide positions where the transitions occurred. Red rhomboids are the hypothetical haplotypes not detected in the analyzed sample (marked with mv). The sizes of the circles are proportional to the number of detected haplotypes as depicted in the legend. The geographical origin of the samples is presented in the legend.

Figure S20.

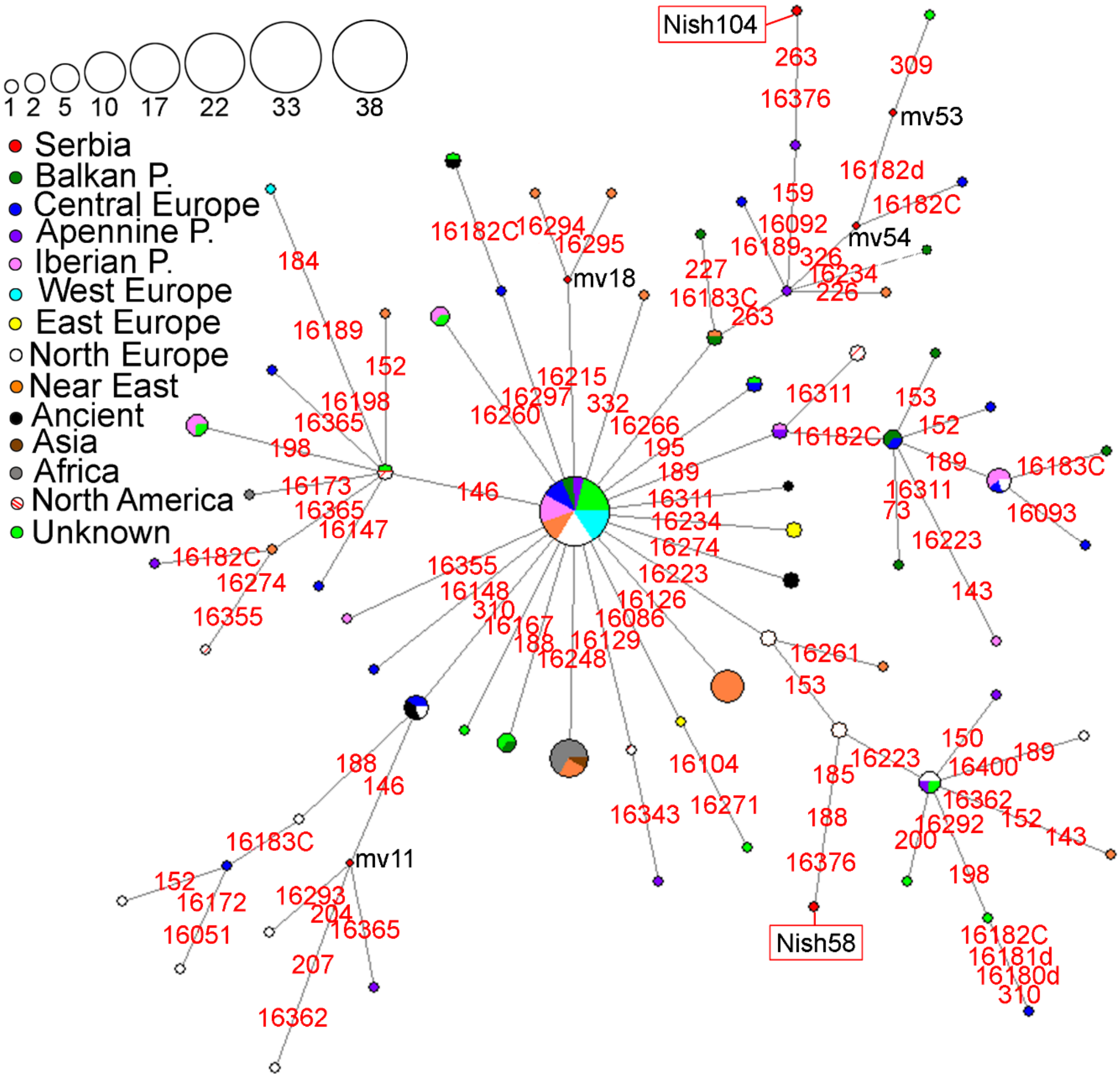

Figure S20. Part of the phylogeographic network for the X2 subhaplogroup based on 458 HVS-I/HVS-II haplotypes detected in 969 individuals which represents the X2n subclade and other X2 subclades that cannot be classified based solely on HVS-I/HVS-II data. Differences from rCRS are marked with red numbers representing nucleotide positions where the transitions occurred. Red rhomboids are the hypothetical haplotypes not detected in the analyzed sample (marked with mv). The sizes of the circles are proportional to the number of detected haplotypes as depicted in the legend. The geographical origin of the samples is presented in the legend.

References:

1. Modi, A.; Nesheva, D.; Sarno, S.; Vai, S. Ancient human mitochondrial genomes from Bronze Age Bulgaria: new insights into the genetic history of Thracians. **2019**, *9*, 5412, doi:10.1038/s41598-019-41945-0.
2. Haak, W.; Lazaridis, I.; Patterson, N.; Rohland, N.; Mallick, S.; Llamas, B.; Brandt, G.; Nordenfelt, S.; Harney, E.; Stewardson, K.; et al. Massive migration from the steppe was a source for Indo-European languages in Europe. *Nature* **2015**, *522*, 207-211, doi:10.1038/nature14317.
3. Neparaczki, E.; Kocsy, K.; Toth, G.E.; Maroti, Z.; Kalmar, T.; Bihari, P.; Nagy, I.; Palfi, G.; Molnar, E.; Rasko, I.; et al. Revising mtDNA haplotypes of the ancient Hungarian conquerors with next generation sequencing. *Plos One* **2017**, *12*, e0174886, doi:10.1371/journal.pone.0174886.
4. Vai, S.; Brunelli, A.; Modi, A.; Tassi, F.; Vergata, C.; Pilli, E.; Lari, M.; Susca, R.R.; Giostra, C.; Baricco, L.P.; et al. A genetic perspective on Longobard-Era migrations. *Eur. J. Hum. Genet.* **2019**, *27*, 647-656, doi:10.1038/s41431-018-0319-8.
5. Allentoft, M.E.; Sikora, M.; Sjogren, K.G.; Rasmussen, S.; Rasmussen, M.; Stenderup, J.; Damgaard, P.B.; Schroeder, H.; Ahlstrom, T.; Vinner, L.; et al. Population genomics of Bronze Age Eurasia. *Nature* **2015**, *522*, 167-172, doi:10.1038/nature14507.
6. Tomory, G.; Csanyi, B.; Bogacsi-Szabo, E.; Kalmar, T.; Czibula, A.; Csosz, A.; Priskin, K.; Mende, B.; Lango, P.; Downes, C.S.; et al. Comparison of maternal lineage and biogeographic analyses of ancient and modern Hungarian populations. *Am J Phys Anthropol* **2007**, *134*, 354-368, doi:10.1002/ajpa.20677.
7. Geary, P. *The Myth of Nations: The Medieval Origins of Europe*; Princeton University Press: Princeton, 2003.
8. Pfeiffer, H.; Brinkmann, B.; Huhne, J.; Rolf, B.; Morris, A.A.; Steighner, R.; Holland, M.M.; Forster, P. Expanding the forensic German mitochondrial DNA control region database: genetic diversity as a function of sample size and microgeography. *Int J Legal Med* **1999**, *112*, 291-298.
9. Davidovic, S.; Malyarchuk, B.; Grzybowski, T.; Aleksic, J.M.; Derenko, M.; Litvinov, A.; Rogalla-Ladniak, U.; Stevanovic, M.; Kovacevic-Grujicic, N. Complete mitogenome data for the Serbian population: the contribution to high-quality forensic databases. *Int J Legal Med* **2020**, *134*, 1581-1590, doi:10.1007/s00414-020-02324-x.
10. Zgonjanin, D.; Veselinovic, I.; Kubat, M.; Furac, I.; Antov, M.; Loncar, E.; Tasic, M.; Vukovic, R.; Omorjan, R. Sequence polymorphism of the mitochondrial DNA control region in the population of Vojvodina Province, Serbia. *Legal Med* **2010**, *12*, 104-107, doi:10.1016/j.legalmed.2009.10.007.
11. Harvey, M.; Gordon, K.; Owens, K.; Lee, M.; King, M.-C. MtDNA sequences from Balkan populations. *NCBI GenBank - Unpublished* **2000**, doi:https://www.ncbi.nlm.nih.gov/nuccore/?term=MtDNA+sequences+from+Balkan+populations.
12. Scorrano, G.; Finocchio, A.; Angelis, F.D.; Martínez-Labarga, C.; Šarac, J.; Contin, I.; Scano, G.; Novokmet, N.; Frezza, D.; Rickards, O. The genetic landscape of Serbian populations through mitochondrial DNA sequencing and non-recombining region of the Y chromosome microsatellites. *Coll. Antropol.* **2017**, *41*, 275-296.

13. Haak, W.; Balanovsky, O.; Sanchez, J.J.; Koshel, S.; Zaporozhchenko, V.; Adler, C.J.; Der Sarkissian, C.S.; Brandt, G.; Schwarz, C.; Nicklisch, N.; et al. Ancient DNA from European early neolithic farmers reveals their near eastern affinities. *Plos Biol* **2010**, *8*, e1000536, doi:10.1371/journal.pbio.1000536.
14. Alt, K.W.; Knipper, C.; Peters, D.; Muller, W.; Maurer, A.F.; Kollig, I.; Nicklisch, N.; Muller, C.; Karimnia, S.; Brandt, G.; et al. Lombards on the Move - An Integrative Study of the Migration Period Cemetery at Szolad, Hungary. *Plos One* **2014**, *9*, e110793, doi:10.1371/journal.pone.0110793.
15. Furtwangler, A.; Rohrlach, A.B.; Lamnidis, T.C.; Papac, L.; Neumann, G.U.; Siebke, I.; Reiter, E.; Steuri, N.; Hald, J.; Denaire, A.; et al. Ancient genomes reveal social and genetic structure of Late Neolithic Switzerland. *Nat. Commun.* **2020**, *11*, 1915, doi:10.1038/s41467-020-15560-x.
16. Carmi, S.; Hui, K.Y.; Kochav, E.; Liu, X.; Xue, J.; Grady, F.; Guha, S.; Upadhyay, K.; Ben-Avraham, D.; Mukherjee, S.; et al. Sequencing an Ashkenazi reference panel supports population-targeted personal genomics and illuminates Jewish and European origins. *Nature Communications* **2014**, *5*, 4835, doi:10.1038/ncomms5835.
17. Xue, J.; Lencz, T.; Darvasi, A.; Pe'er, I.; Carmi, S. The time and place of European admixture in Ashkenazi Jewish history. *PLoS genetics* **2017**, *13*, e1006644, doi:10.1371/journal.pgen.1006644.
18. Yardumian, A.; Schurr, T.G. The Geography of Jewish Ethnogenesis. *J. Anthropol. Res.* **2019**, *75*, 206-234, doi:10.1086/702709.
19. Brandt, G.; Haak, W.; Adler, C.J.; Roth, C.; Szecsenyi-Nagy, A.; Karimnia, S.; Moller-Rieker, S.; Meller, H.; Ganslmeier, R.; Friederich, S.; et al. Ancient DNA Reveals Key Stages in the Formation of Central European Mitochondrial Genetic Diversity. *Science* **2013**, *342*, 257-261, doi:10.1126/science.1241844.
20. Juras, A.; Ehler, E.; Chylenski, M.; Pospieszny, L.; Spinek, A.E.; Malmstrom, H.; Krzewinska, M.; Szostek, K.; Pasterkiewicz, W.; Florek, M.; et al. Maternal genetic origin of the late and final Neolithic human populations from present-day Poland. *Am J Phys Anthropol* **2021**, *176*, 223-236, doi:10.1002/ajpa.24372.
21. Oversti, S.; Majander, K.; Salmela, E.; Salo, K.; Arppe, L.; Belskiy, S.; Etu-Sihvola, H.; Laakso, V.; Mikkola, E.; Pfrengle, S.; et al. Human mitochondrial DNA lineages in Iron-Age Fennoscandia suggest incipient admixture and eastern introduction of farming-related maternal ancestry. *Sci. Rep.* **2019**, *9*, 16883, doi:10.1038/s41598-019-51045-8.
22. Klunk, J.; Duggan, A.T.; Redfern, R.; Gamble, J.; Boldsen, J.L.; Golding, G.B.; Walter, B.S.; Eaton, K.; Stangroom, J.; Rouillard, J.M.; et al. Genetic resiliency and the Black Death: No apparent loss of mitogenomic diversity due to the Black Death in medieval London and Denmark. *Am J Phys Anthropol* **2019**, *169*, 240-252, doi:10.1002/ajpa.23820.
23. Juras, A.; Chylenski, M.; Ehler, E.; Malmstrom, H.; Zurkiewicz, D.; Wlodarczak, P.; Wilk, S.; Peska, J.; Fojtik, P.; Kralik, M.; et al. Mitochondrial genomes reveal an east to west cline of steppe ancestry in Corded Ware populations. *Sci. Rep.* **2018**, *8*, 11603, doi:10.1038/s41598-018-29914-5.
24. Matisoo-Smith, E.; Gosling, A.L.; Platt, D.; Kardailsky, O.; Prost, S.; Cameron-Christie, S.; Collins, C.J.; Boocock, J.; Kurumilian, Y.; Guirguis, M.; et al. Ancient mitogenomes of Phoenicians from Sardinia and Lebanon: A story of settlement, integration, and female mobility. *Plos One* **2018**, *13*, e0190169, doi:10.1371/journal.pone.0190169.

25. Chylenski, M.; Ehler, E.; Somel, M.; Yaka, R.; Krzewinska, M.; Dabert, M.; Juras, A.; Marciniak, A. Ancient Mitochondrial Genomes Reveal the Absence of Maternal Kinship in the Burials of Catalhoyuk People and Their Genetic Affinities. *Genes* **2019**, *10*, doi:10.3390/genes10030207.
26. Knipper, C.; Mittnik, A.; Massy, K.; Kociumaka, C.; Kucukkalipci, I.; Maus, M.; Wittenborn, F.; Metz, S.E.; Staskiewicz, A.; Krause, J.; et al. Female exogamy and gene pool diversification at the transition from the Final Neolithic to the Early Bronze Age in central Europe. *P Natl Acad Sci USA* **2017**, *114*, 10083-10088, doi:10.1073/pnas.1706355114.
27. Modi, A.; Vizzari, M.T.; Catalano, G.; Boscolo Agostini, R.; Vai, S.; Lari, M.; Vergata, C.; Zaro, V.; Liccioli, L.; Fedi, M.; et al. Genetic structure and differentiation from early bronze age in the mediterranean island of sicily: Insights from ancient mitochondrial genomes. *Front. Genet.* **2022**, *13*, 945227, doi:10.3389/fgene.2022.945227.
